# As our population changes, so must we: how race and ethnicity data merging has evolved over time in the Wisconsin Administrative Data Core

**DOI:** 10.23889/ijpds.v9i5.2881

**Published:** 2024-09-18

**Authors:** Krista Bryz-Gornia, Katie Thornton

**Affiliations:** 1 Institute for Research on Poverty- University of Wisconsin-Madison

The Institute for Research on Poverty (IRP), centered at the University of Wisconsin-Madison in Wisconsin, USA, began collaboration with various Wisconsin state agencies in 1985 to link data between disparate state agencies. These initial efforts have since evolved into the Wisconsin Administrative Data Core (WADC) which is an individual-level population dataset connecting extensive administrative data spanning decades of Wisconsin social services program history. Since its purpose is to facilitate evidence-driven policymaking and support program evaluation, it is essential that our data linking efforts accurately represent our Wisconsin population.

Since its inception, the WADC has adapted to changes in how race and ethnicity are collected and reported. While Wisconsin has a racial makeup that is generally less diverse than the national population, the number of non-white and multi-racial identifying residents has increased over time. Furthermore, varying state agency demographic collection and reporting methods have fueled the need for our linking methods to change, too.

IRP initially implemented standard data linkage methods using a mixture of probabilistic and deterministic matching of personal identifying information. But as the racial composition of Wisconsin residents changed over time, our linking assumptions induced implicit biases. In response, we updated our matching algorithm to account for the cultural nuances in how personally identifying information is assigned and reported within various racial and ethnic subgroups. Furthermore, we prioritized values from data sources that were self-reported rather than assigned from a third-party. These methodological modifications have improved the WADC’s reliability and representativeness of the population.

